# Supplementary material for: Pb Content, Risk Level and Primary-Source Apportionment in Wheat and Rice Grains in the Lihe River Watershed, Taihu Region, Eastern China
Source: Int J Environ Res Public Health. 2021 Jun 9;18(12):6256. doi: 10.3390/ijerph18126256 (PMC8295996; doi:10.3390/ijerph18126256)
Supplement: Supplementary file 1 [file ijerph-18-06256-s001.zip › ijerph-1192829-supplementary.pdf]

Pb content, risk level and primary-source apportionment in wheat and rice grains in the Lihe River watershed, Taihu region

Contents

Figure S1. Location of the study area and distribution of sampling points.

Figure S2. Relationships between $^{207}Pb/^{206}Pb$ and $^{208}Pb/^{206}Pb$ for soils and possible contamination sources. SM = suspended matter in irrigation water; filtered water = filtered irrigation water.

Figure S3. Pb isotopic ratios in dustfall and its Pb sources.

Table S1. Mean value of calculation parameters for daily average exposure of Pb
Table S2. Division of health risk grade.
Table S3. Values of physicochemical properties in wheat and rice soil.

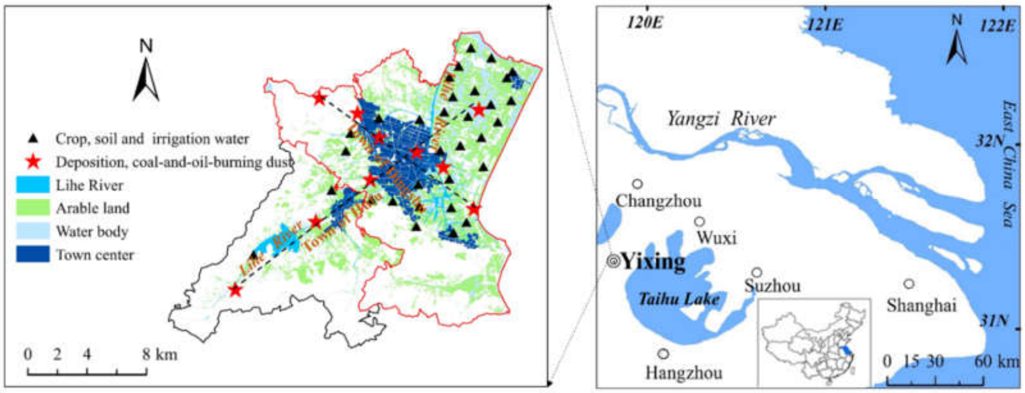

Figure S1. Location of the study area and distribution of sampling points.

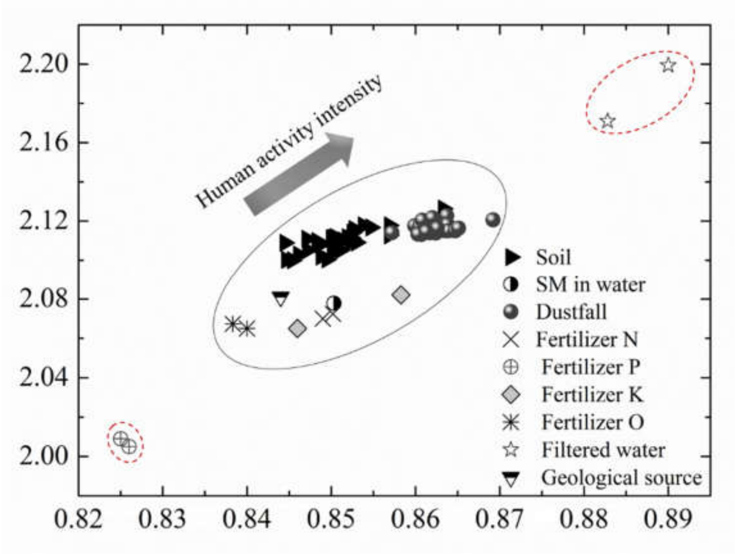

Figure S2. Relationships between $^{207}Pb/^{206}Pb$ and $^{208}Pb/^{206}Pb$ for soils and possible contamination sources. SM = suspended matter in irrigation water; filtered water = filtered irrigation water.

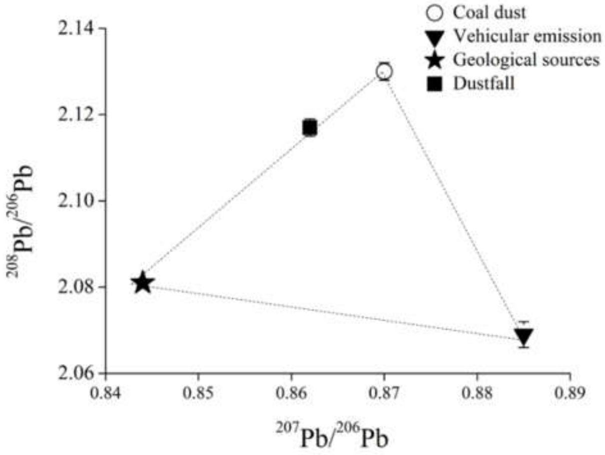

Figure S3 Pb isotopic ratios in dustfall and its Pb sources.

Table S1. Mean value of calculation parameters for daily average exposure of Pb

Parameter	Meaning	Unit	Child	Adult
C_w	Pb concentration in wheat grains	$mg·kg^{-1}$	**0.539**	**0.539**
C_r	Pb concentration in rice grains	$mg·kg^{-1}$	**0.447**	**0.447**
BW	Average bodyweight	kg	**17.6**	**67.6**
EF	Exposure frequency	$d·a^{-1}$	365	365

ED	Exposure duration	a	6	70
AT	Average exposure time	d	365×ED	365×ED
CF	Unit conversion	$kg \cdot mg^{-1}$	1×10^{-6}	1×10^{-6}
$IngR_w$	Ingestion rate of wheat	$mg \cdot d^{-1}$	**0.039**	**0.101**
$IngR_r$	Ingestion rate of rice	$mg \cdot d^{-1}$	**0.091**	**0.234**

Note: the data in bold are the results of interview survey or test, and other data are provided by US EPA.

Table S2. Division of health risk grade.

Risk grade	Low	Medium-low	Medium	Next higher	high
Range of HQ	1<HQ≤1.5	1.5 < HQ≤ 2	2< HQ≤2.5	2.5 < HQ≤3	3< HQ

Table S3. Values of physicochemical properties in wheat and rice soil.

Soil properties	Soil type	Range	Mean	SD	CV
pH	Wheat soil	4.85-7.45	5.81	0.69	11.88
	Rice soil	4.99-7.59	6.00	0.76	12.61
CEC（$cmol \cdot kg^{-1}$）	Wheat soil	11.76-22.99	18.41	2.60	14.11
	Rice soil	14.78-23.79	18.31	2.88	15.74
EC（$\mu s \cdot cm^{-1}$）	Wheat soil	672-3050	1592.25	648.13	40.71
	Rice soil	695-4090	1934.78	866.24	44.77
TOC	Wheat soil	1.84-5.35	3.58	0.94	26.20
	Rice soil	0.97-6.62	3.45	1.45	42.11
Mean particle size	Wheat soil	8.45-52.06	24.37	8.37	34.41
	Rice soil	7.46-29.25	17.73	5.41	30.54

Note: “SD” represent “standard deviation”; “CV” represent “coefficient of variation”.
